# A novel approach to Fabry–Pérot-resonance-based lens and demonstrating deep-subwavelength imaging

**DOI:** 10.1038/s41598-020-67409-4

**Published:** 2020-07-01

**Authors:** Md. Anzan-Uz-Zaman, Kyungjun Song, Duck-Gyu Lee, Shin Hur

**Affiliations:** 1 Department of Nature-Inspired Nano Convergence Systems, Korea Institute of Machinery and Materials, 156 Gajeongbuk-Ro, Daejeon, 34103 Republic of Korea; 2 Nano-Mechatronics, University of Science and Technology, 217 Gajeong-ro, Daejeon, 34103 Republic of Korea; 3 Department of Mechanical Engineering, Pusan National University, 63-2 Geumjeong-Ku, Busan, 46241 Republic of Korea

**Keywords:** Mechanical engineering, Acoustics

## Abstract

During our research, we explored a novel way to represent subwavelength imaging and derived a transmission equation to explicate the FP (Fabry**–**Pérot) resonance phenomena. Subsequently, using analysis and observation, we performed deep-subwavelength imaging. Both numerically and experimentally, imaging with super-resolution was achieved at deep subwavelength scale of λ/56.53 with a lens thickness 212 mm. Our results also showed that by increasing lens thickness, higher resolution can be achieved. Moreover, via a single source study, we showed the full width at half maximum range and predicted the size of smallest detectable object. We also observed that with a greater lens thickness, finer features could be detected. These findings may open a new route in near-field imaging for practical applications such as biometric sensors, ultrasonic medical equipment, and non-destructive testing.

## Introduction

In 1873, Ernst Abbe made an observation that the minimum resolvable distance for a wave is $\lambda /\left( {2 NA} \right),$ where NA is the numerical aperture, which is equal to ${\text{ n}} \cdot \sin \theta$^1^. This constraint for imaging is known as the Abbe diffraction limit. Generally, by considering the numerical aperture as roughly equal to one, the diffraction limit is given as half of the wavelength. Therefore, owing to this diffraction limit, with conventional materials available in nature, an image resolution beyond λ/2 cannot be attained. However, with the advent of metamaterials over the last two decades, artificial materials with novel properties^2–8^ have been demonstrated. The concept of metamaterials dates back to more than 50 years ago, when it was discovered by the Russian physicist Victor Veselago during his prominent work^9^, in which he proposed a method to achieve a negative refractive index by demonstrating negative permittivity and permeability theoretically. However, obtaining the new attributes of metamaterial have inspired researchers to overcome the diffraction limit. In 2000, Pendry, for first time, successfully demonstrated a superlens numerically using a negative refractive index^10^. Several other reports^11–16^ can be found on using a negative index material (NIM), an ɛ-negative material (ENG), or a µ-negative material (MNG) to overcome the diffraction limit for electromagnetic waves. These reports show that a NIM is not an essential condition for achieving subwavelength imaging. In the acoustic regime as well, a single negative material, such as the ρ-NG metamaterial^17–19^, can be used to realize subwavelength imaging. Double negativity has also been used for this purpose^20, 21^. Even a positive only metalens^22^, which is based on Fano resonance, has been observed. Different approaches, such as time reversal^23–26^ and Bragg scattering^27–29^ (in phononic crystal) have been employed to attain subwavelength resolutions. Gradient Index metasurface and super-oscillation technique may also be employed to achieve such kind of resolution^30,31^. It should be mentioned here that the latter technique can be used for imaging in far-field. Given a highly anisotropic medium, several metamaterials have been proposed for subwavelength imaging^32,33^ which may applicable in only very close proximity of the lens. Metamaterials with higher refractive indices^34,35^, i.e., ${\text{n}} \gg 1$, and/or the guided mode^36^ have been reported to break the diffraction limit. In the year of 2011, Zhu et al.^37^ provided the eminent transmission equation using the resemblance of acoustic wave to light through 1D slit and demonstrated deep-subwavelength imaging with the highest resolution of λ/50 by creating a highly anisotropic medium and using Fabry–Pérot (FP) resonance. However, FP-based lenses come with a set of limitations, such as those due to resonance; further, the imaging frequency bandwidth is very narrow, and the resonance frequency depends on the lens thickness. Furthermore, due to the dependence on thickness, the lens becomes bulky for low-frequency imaging. To overcome this, another type of anisotropic acoustic metamaterial has been reported based on the resonant tunneling method^38–40^. The frequency was made independent of the thickness using the Bloch wave resonance, which can be achieved by diameter modulation. Lenses designed using metamaterials with near-zero^41^ or zero mass^42^ have also shown the property of imaging frequency being independent of lens thickness. Although FP-based lenses may not be suitable at low frequencies, they are a candidate for pragmatic use because they can be used in high-frequency applications. Moreover, unlike the resonant tunneling lens, the analytical and experimental frequencies of FP-based lens are similar, which is expedient for practical applicability. The main principle behind an FP lens is the full transmission of both the evanescent and propagating waves at the FP resonance. As evanescent waves contain higher parallel wave momentum, which holds infinitesimal feature information, retrieving them at the image plane makes deep-subwavelength imaging possible. Unlike FP lens, excluding propagating wave component, only evanescent wave based lens was also demonstrated by manipulating trapped resonances and Bragg scattering^43^. However, several reports^44–47^ on various aspects of FP resonance based lens can be observed based on earlier theory. A work on optimization of the design parameters of holey structured metamaterial lens and experimental demonstration of subwavelength resolution (λ/25) in the ultrasonic regime was studied^46^. But, the optimization was mostly based on numerical data and not directly related with the assumption for which the transmission equation remains valid and the experiments were carried out in water. On the contrary, at the current work, the resolution was enhanced in air medium by only manipulating the lens thickness (h) while maintaining high ratio of wavelength to hole feature size for keeping the transmission equation valid. Moreover, in present paper, unlike the conventional theory^37^, we used a novel approach named as three-medium model which is not adapted from the EM counterpart, rather from acoustic regime and, thus may provide a fresh perspective to derive the transmission equation of FP-resonance-based lens. We were also able to go beyond the existing highest resolution (λ/50) for imaging in acoustic case which was persisted for almost last one decade and achieved the new highest resolution as λ/56.53 at subwavelength scale.

However, due to the capability of deep subwavelength imaging of FP lens and thus envisaging the applicability to biometrics, such as in ultrasonic fingerprint sensors, we were interested in exploring and understanding each minute detail of the lens. Thus, this study explores a novel way to elucidate the FP based lens which might be easier to understand the phenomena in association of conventional theory^37^ and may provide fresh perspective in the field. Finally, we hope that the findings presented here will facilitate researchers to apply the lens for practical purposes.

## Results

### Exploration of the novel theory

**Figure 1:**
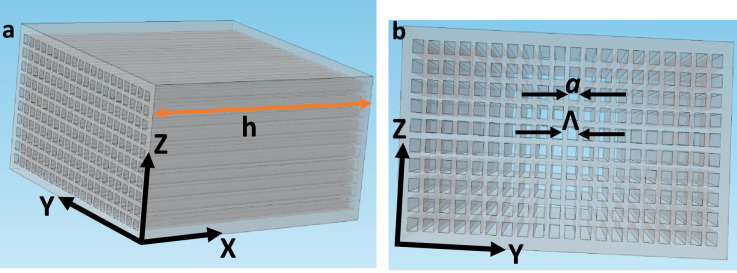
Fabry-Pérot resonance based lens with square shaped holes. Here *a* = feature size of holes, Λ = periodicity and h = thickness. (**a**) Side view. (**b**) Front view of the lens.

Figure 1 shows a holey structured FP resonance based lens in conventional Cartesian co-ordinate system consisting of square holes with a feature size ‘*a*’. The holes are placed along Y and Z axes with a periodicity ‘Λ’. The thickness of the lens is h. In our novel approach, a three (effective) medium concept is applied to a single aperture of the lens. According to Blackstock^48^, for normal incidence, the transmission coefficient can be given as

1 $$T = \frac{2}{{(1 + Z_{1} /Z_{3} )\cos k_{2} l + j(Z_{2} /Z_{3} + Z_{1} /Z_{2} )\sin k_{2} l}}$$

where Z_1_, Z_2_, and Z_3_ = impedances of first, second and third medium, *l* = length of the second medium, *k*_*2*_ = wave number of the incident wave in the second medium.

**Figure 2:**
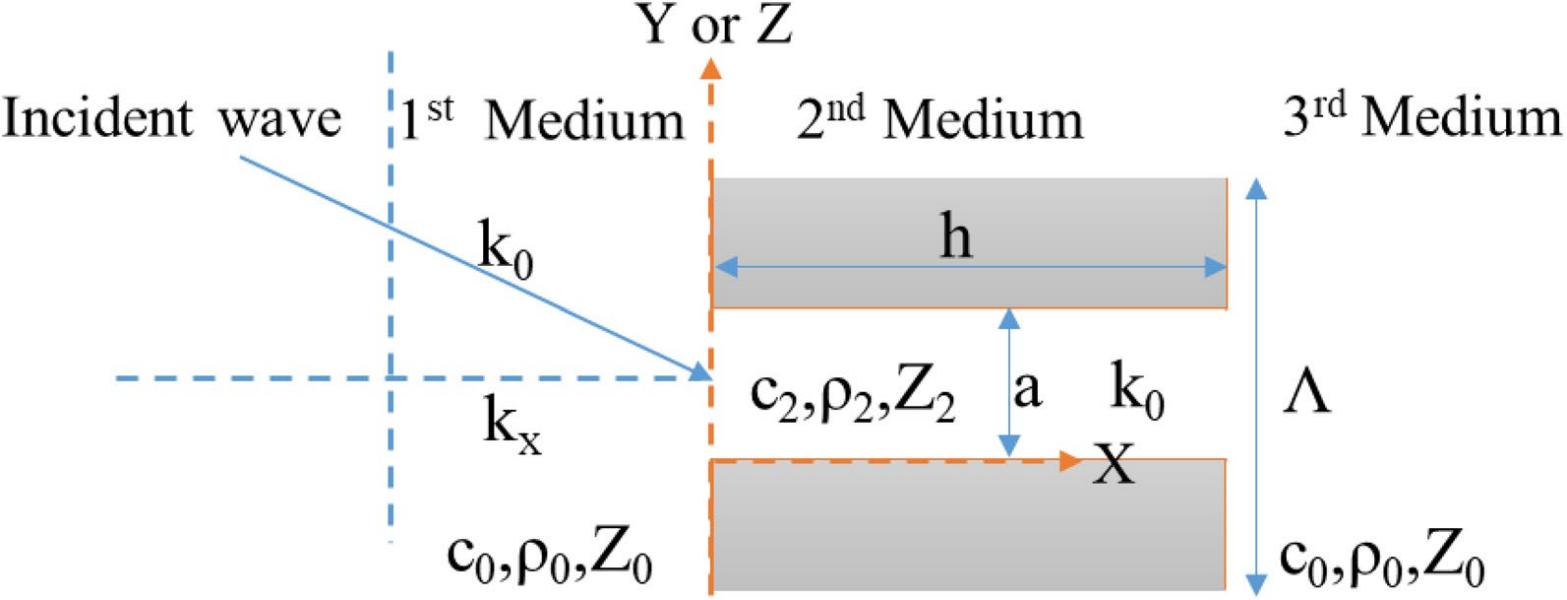
2D conceptual representation of a wave incident to a single hole in the lens.

As shown in Fig. 2, the first and third media are identical. The second medium consists of a holey-structured metamaterial filled with the same fluid as in the first and third media. According to Fig. 2, a sound wave of wavelength λ is obliquely incident on a hole. The wavenumber of the wave is ${\text{k}}_{0}$ and the normal component of the wavenumber is ${\text{k}}_{{\text{x}}}$. The parallel component can be given as ${\text{k}}_{\parallel } = \sqrt {{\text{k}}_{{\text{y}}}^{2} + {\text{k}}_{{\text{z}}}^{2} }$. Therefore, the incident wavenumber can be expressed as ${\text{k}}_{0} = \sqrt {{\text{k}}_{{\text{x}}}^{2} + k_{\parallel }^{2} }$. The sound velocity, density, and impedance of the first and third media are $c_{0} ,\rho_{0} , {\text{and }}Z_{0}$, respectively. Accordingly, the normal component of the velocity $\left( {c_{0} } \right)$ is $c_{x} .$ Therefore, for the oblique incident wave, the impedance in the normal direction can be realized with the following equation: $Z_{0x} = \rho_{0} c_{x} = \rho_{0} \left( {\omega /{\text{k}}_{{\text{x}}} } \right) = \omega \rho_{0} /\sqrt {{\text{k}}_{0}^{2} - k_{\parallel }^{2} } .$ Intuitively, it may be assumed that if ${\uplambda } \gg a$, then the velocity of the sound in the second medium, i.e., inside the hole, can be given as $c_{2} = c_{0}$, which implies that the wavenumber inside the hole is ${\text{k}}_{hole} = {\text{k}}_{0} .$ Therefore, according to Zhu et al.^37^, the effective impedance of the second medium can be represented as ${\text{Z}}_{2} = {\text{Z}}_{{{\text{fluid}}}} /\left| {S_{00} } \right|^{2}$, where ${\text{Z}}_{{{\text{fluid}}}} = {\text{Z}}_{0} = \rho_{0} {\text{c}}_{0} = \omega \rho_{0} /{\text{k}}_{0}$ and $S_{00} = a/\Lambda$. As the length of the hole is *h*, according to Eq. (1), the transmission equation can be obtained as

2 $$T = \frac{2}{{2\cos k_{0} h + j(Z_{2} /Z_{0x} + Z_{0x} /Z_{2} )\sin k_{0} h}}$$

Using Eq. (2), the tunneling condition can be expressed as

3 $$\left| T \right| = 1,\quad for \,\,\,k_{0} h = {\text{m}}\pi ,{ }\,{\text{where m}} = {\text{any integer}}$$

Equation (3) also shows that the eminent FP resonant condition for tunneling can be obtained if the hole thickness is an integer multiple of a half-wavelength. To derive the resonance frequencies from Eq. (3), we may write

4 $$\lambda = \frac{2h}{m}$$

**Figure 3:**
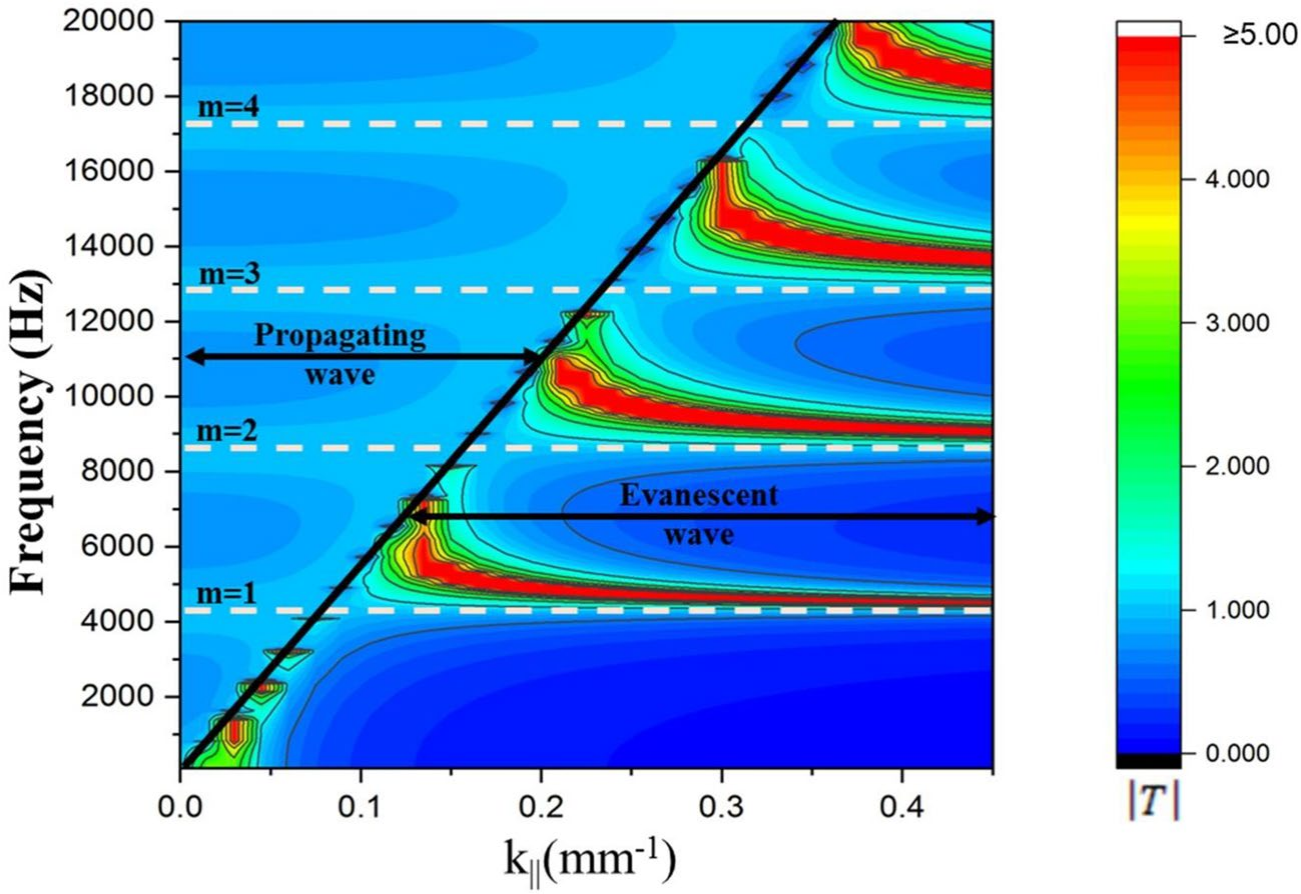
Contour plot of transmission coefficient $\left| T \right|$ over a frequency range of 100–20,000 Hz for a lens with parameters $a = 1.0 \,\,{\text{mm}}, \,\,\Lambda = 1.5\,\,{\text{ mm}},\,\,{\text{ and}}\,\,\, h = 40.0\,\, {\text{mm}}$.

Analysis based on Eq. (2) validates the full transmission of the evanescent and propagating waves at the FP resonance, which can be observed in Fig. 3. To obtain the contour plot, air was considered as the fluid, and the velocity ($$c_{0} )$$ and density ($$\rho_{0}$$) of air were given as 343 m/s and 1.25 kg/m^3^, respectively. The length of the square hole (*h*), feature size (*a*), and periodicity (*Λ*) were kept as 40.0, 1.0, and 1.5 mm respectively. Flat uniform dispersion lines for both the evanescent and propagating waves are seen for *m* = 1–4, which corresponds to 4,287.5, 8,575.0, 12,862.5, and 17,150.0 Hz. The transmission coefficient on these lines is exactly one, indicating that full transmission will occur at the FP resonance frequencies. Further, and more significantly, as both the evanescent and propagating waves will be tunneled, the diffraction limit may be transcended. However, if we closely observe the contour plot, may realize that except tunneling region, the transmission coefficient for other region differs from the existing model. This may happen due to using different definitions of wavefunction. In future, based on objectives, further study can be conducted on this regard. However, at this study, the transmission characteristics of FP-resonance-based lens has been explored and elucidated from a different viewpoint through a novel approach named as three-medium model which may provide a fresh perspective in acoustic regime. Next, the analytical model (as shown in Fig. 3) is substantiated by numerical and experimental analyses.

### Numerical study

**Figure 4:**
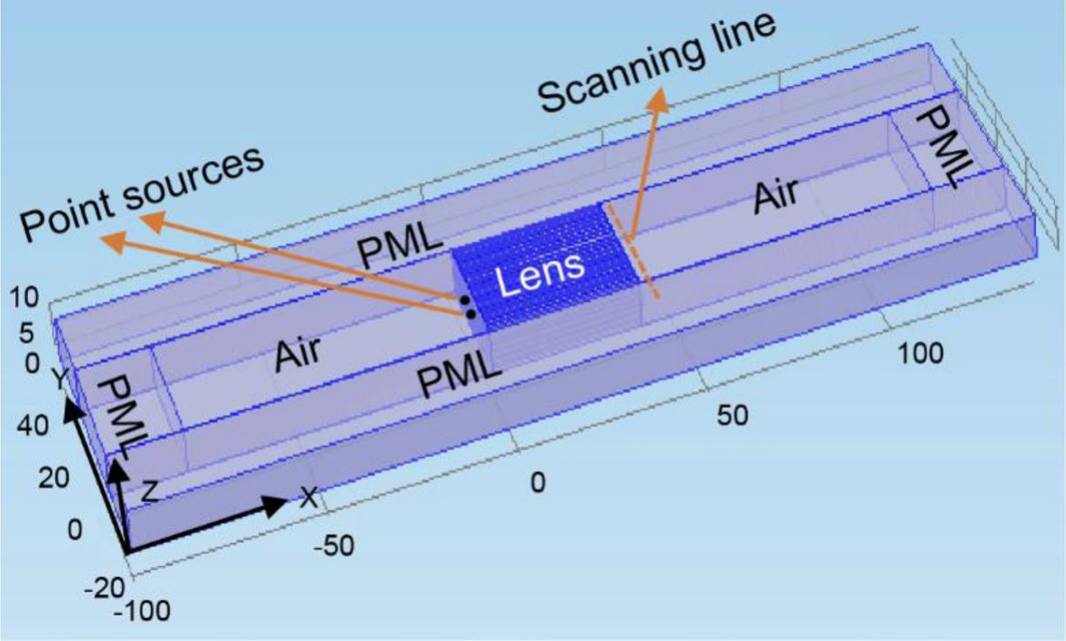
Numerical model created in the COMSOL Multiphysics software with lens parameters $a = 1.0{\text{ mm}},{ }\Lambda = 1.5{\text{ mm}},{\text{ and h}} = 40.0{\text{ mm}}$.

For numerical analysis, the finite element method (FEM)-based commercial software COMSOL Multiphysics was employed. Two monopole point sources were positioned in front of the lens and the distance between the lens and the sources was 0.5 mm. The distance between the sources was maintained at 7.5 mm. The flow rate of the sources was 0.001 m^3^/s. As shown in Fig. 4, scanning was performed behind the lens, at a distance of 0.5 mm. The height (in the z-direction) of the sources and the scanning line was the same.

**Figure 5:**
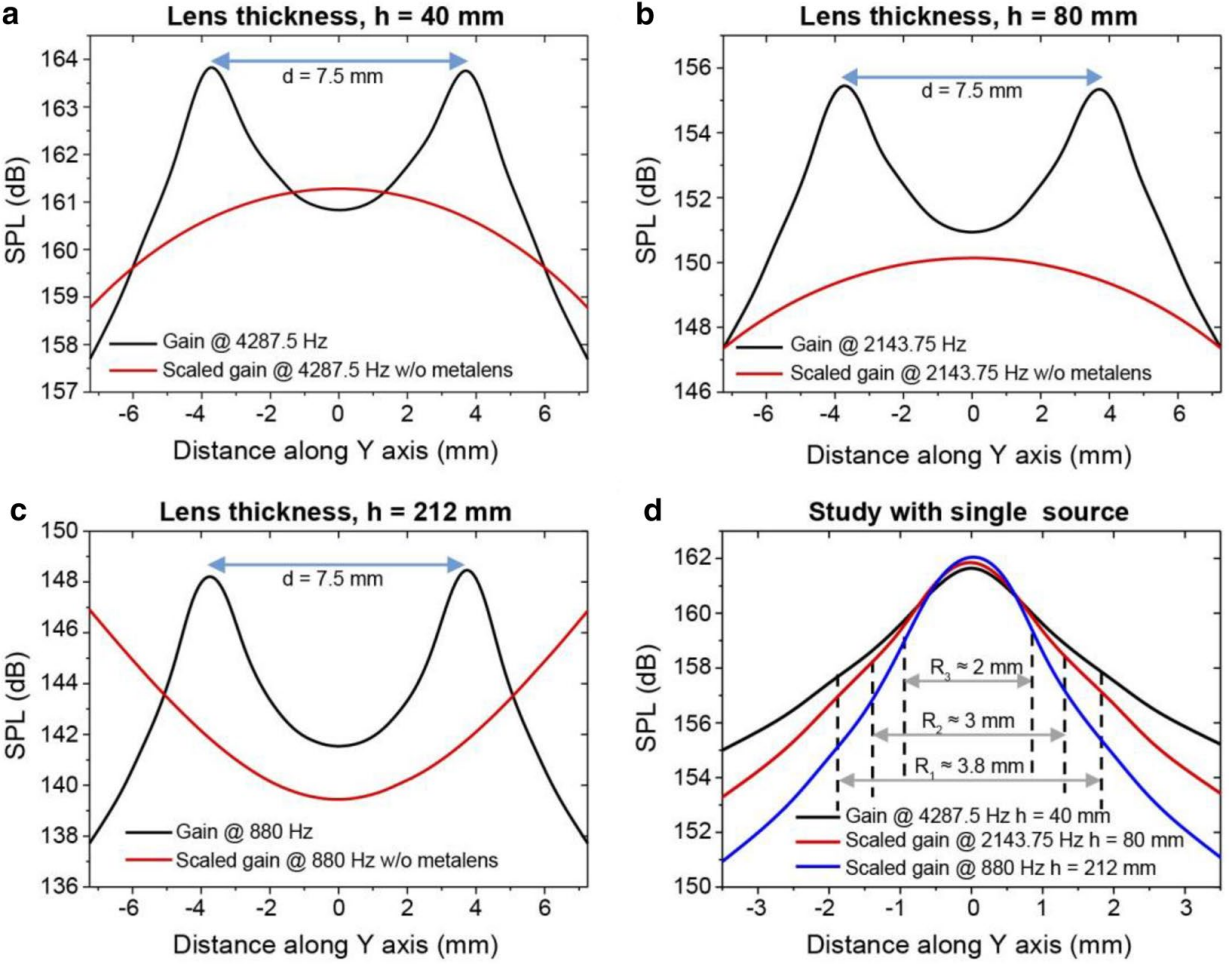
Numerical results from the COMSOL Multiphysics simulation. Distance between the two monopole point sources = d. (**a**) Results obtained with lens thickness *h* = 40 mm; without the lens, the data were scaled by adding 22 dB to the original data. (**b**) Results obtained with lens thickness *h* = 80 mm; without the lens, the data were scaled by adding 67 dB to the original data. (**c**) Results obtained with lens thickness *h* = 212 mm; without the lens, the data were scaled by adding 125 dB to the original data. (**d**) Data obtained with a single monopole source using metalenses with thicknesses *h* = 40, 80, and 212 mm. Data with lens thicknesses *h* = 80 and 212 mm were scaled by adding 8 and 15 dB, respectively. R1, R2, and R3 are the ranges for the full width at half maximum (FWHM) 3 dB below the peak.

Initially, a lens thickness *h* of 40 mm was considered; according to Eq. (4), the lowest frequency was 4,287.5 Hz, which corresponds to λ = 80 mm at *m* = 1. As shown in Fig. 5a, two sharp peaks can be directly discerned near the source locations at $\pm$ 3.75 mm. Note that the distance between the sources was λ/10.76, which is far below the diffraction limit. However, if we observe the scaled line without the metalens, we will not find any sign of the sources, i.e., the source locations cannot be detected without the metalens. Furthermore, the data without the metalens were scaled by adding a 22 dB SPL (sound pressure level) to represent the original data within the range of the data with the metalens. Finally, comparing the two data sets (with and without that metalens) revealed that an image with subwavelength resolution has been realized with the proposed lens in Fig. 4. Next, Eq. (4) reveals that increasing the lens thickness decreases the lowest imaging frequency at *m* = 1, which in turn can increase the resolution. Hence, the lens thickness was increased to 80 and 212 mm while keeping all the other parameters constant. As shown by the results in Fig. 5b,c, at both 2,143.75 and 808.0 Hz, the same distance, d, of 7.5 mm can be applied for imaging. Moreover, as the wavelength increases for the same d, the detectable minimum distance for these frequencies are at deep subwavelength scale which are λ/21.33 and λ/56.53, respectively. The numerical resolution depicted in Fig. 5c for a lens thickness *h* = 212 mm is the highest resolution ever reported for subwavelength imaging. However, although an enhancement in resolution may be achieved by increasing the lens thickness, it may cause the lens to become bulky. Therefore, based on requirements, an appropriate lens thickness should be chosen.

We also studied all three lenses with a single source to observe the subwavelength scale of FWHM (full width at half maximum) range of the SPL distribution of sound wave; the results are presented in Fig. 5d. Due to the interference among waves coming from the sources, probably below the FWHM range, the sources cannot be distinguished. Thus, a study with a single source provides insight into the highest possible resolution. Figure 5d reveals that the lowest possible feature size is 3.8, 3.0, and 2.0 mm with lens thicknesses of 40, 80, and 212 mm, respectively. Thus, a greater lens thickness allows a smaller object to be detected; that is, a higher resolution is achieved by increasing the lens thickness.

### Experimental study

**Figure 6:**
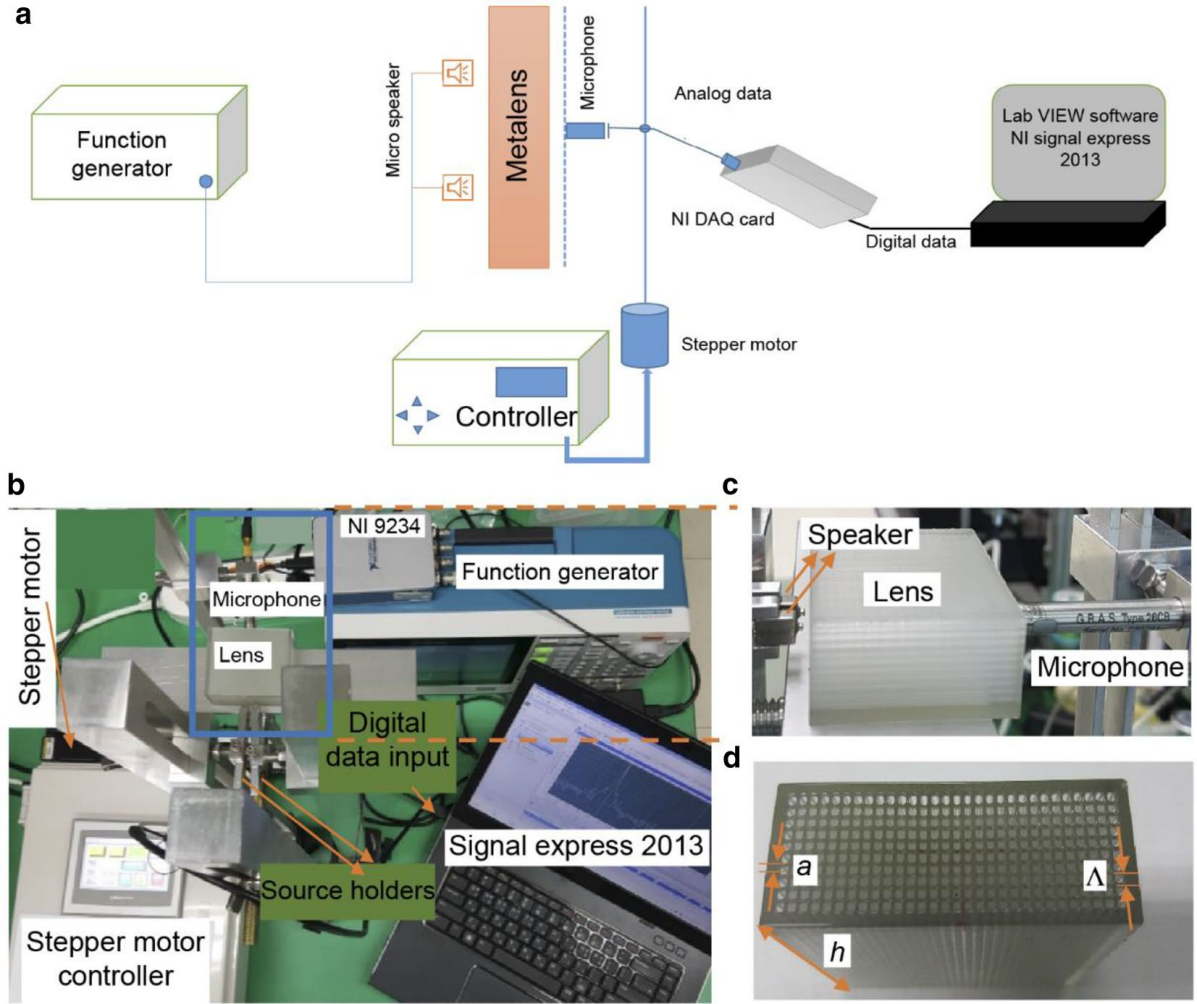
Experimental setup. (**a**) Conceptual diagram of the experimental setup with a metalens and various tools and instruments. (**b**) Actual experimental setup. (**c**) Lateral view of the inset in (**b**) (blue box around the lens and microphone) showing the positions of the speakers, lens, and microphone in close proximity. (**d**) Top view of the fabricated lens.

The conceptual experimental setup is depicted in Fig. 6a to aid in understanding the basic working procedure of the experiment, as shown in Fig. 6b. All the lenses were fabricated using 3D printing technology, which made the fabrication process very convenient. Initially, we fabricated a metalens with the same parameters as those considered for the numerical analysis in Fig. 3, i.e., $a = 1.0 \,\,{\text{mm}}, \Lambda = 1.5 \,\,{\text{mm}},\,\,{\text{ and }}\,\,h = 40.0\,\,{\text{ mm}}$. As shown in Fig. 6c, two micro-speakers are placed in front of the lens in close proximity to retrieve the evanescent wave and the distance between the speakers, d, in the y-direction is set as 7.5 mm. The speakers are excited by a function generator and directed along the x-axis. As the speakers are separated by a subwavelength distance in the y-direction, scanning behind the lens along the same direction will indicate whether any imaging with the lens beyond the diffraction limit is possible. Hence, a microphone is positioned behind the lens at the same height as the speakers in the z-direction to obtain image data, as can be seen in Fig. 6c. A wider view of the fabricated lens is given in Fig. 6d.

**Figure 7:**
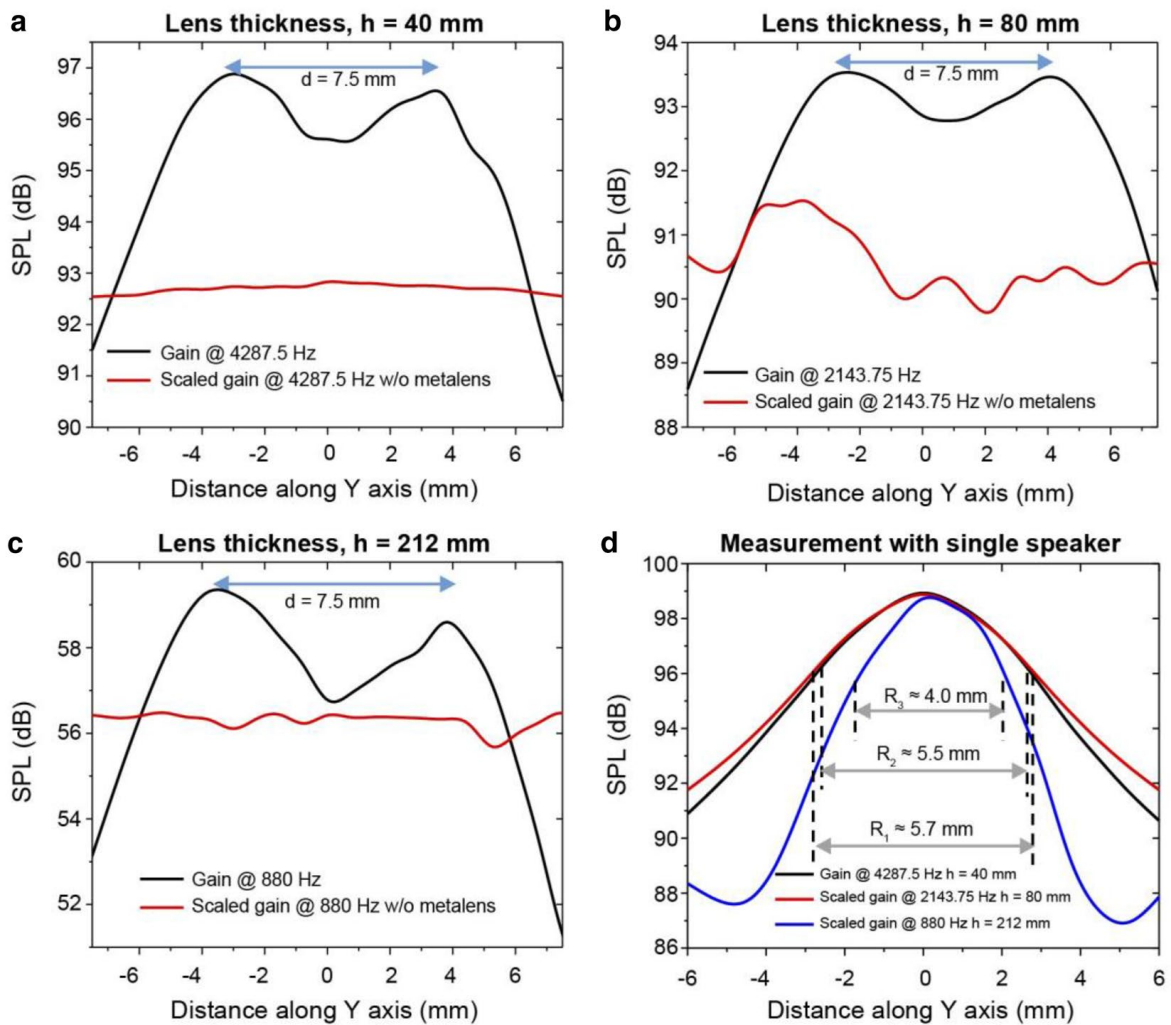
Experimental results. Distance between the speakers = d. (**a**) Results obtained with lens thickness *h* = 40 mm. Without the lens, the data were scaled by adding 15 dB to the original data. (**b**) Results obtained with lens thickness *h* = 80 mm. Without the lens, the data were scaled by adding 23 dB to the original data. (**c**) Results obtained with lens thickness *h* = 212 mm. Without the lens, the data were scaled by adding 5 dB to the original data. (**d**) Data obtained with a single speaker using metalenses with thicknesses *h* = 40, 80, and 212 mm. The data with lens thicknesses *h* = 80 and 212 mm were scaled by adding 4 and 39 dB, respectively. R_1_, R_2_, and R_3_ are the FWHM ranges 3 dB below the peak.

Similar to the FEM simulation, the two sources were positioned at $\pm$ 3.75 mm. According to Ernst Abbe’s observation^1^ for a wave with λ = 80 mm, the sources cannot be detected separately as d (= 7.5 mm) is far below the diffraction limit. This observation agrees with that presented in Fig. 7a. When no lens is used, the two sources are indistinguishable from the graph. However, when a lens with *h* = 40 mm is inserted between the sources and the microphone, two clear peaks of SPL are observed on the opposite side of the lens at the same $\pm$ 3.75 mm distance in the y-direction. This result provides experimental evidence for FP resonance, which has been exploited for subwavelength imaging. In the next step, based on Eq. (4), we increased the lens thickness gradually to determine whether increasing lens thickness can increase the wavelength of the imaging frequency and consequently enhance the resolution. For this, we fabricated two lenses with *h* = 80 and 212 mm. From Eq. (4), for *m* = 1, the lowest possible wavelengths can be deduced as 160 and 424 mm for 2,143.75 and 818.0 Hz, respectively. Figure 7b,c demonstrate that for distance d = 7.5 mm, the image is well resolved for both lens, which indicates resolutions of λ/21.33 and λ/56.53, respectively. A comparison of these results with those of our numerical study, shown in Fig. 5b,c, reveals good consistency, and therefore, our experiment provides evidence of deep-subwavelength imaging with FP resonance. Notably, this study provides experimental evidence of the imaging at deepest subwavelength scale ever reported in acoustic regime: λ/56.53.

As in the numerical analysis, we also performed an experiment with a single source to minimize the possible detectable feature size for our designed lenses. From Fig. 7d, the FWHM ranges R_1_, R_2_, and R_3_ are obtained 5.7, 5.5, and 4.0 mm for lens with thicknesses of 40, 80, and 212 mm, respectively. In comparison with numerical data, the obtained experimental ranges were larger for each lens. Although the experiment was conducted in a big auditorium to minimize the scattering and provide an anechoic chamber environment, it was not possible to neglect all the noise that may be caused in larger FWHM ranges. It should also be referred here that maybe during experiment inherent losses (thermal and viscous) had higher effect on the variation of FWHM range than realized at the simulation. However, similar to numerical results, a greater thickness allows a smaller FWHM range to be achieved. Hence, by increasing the lens thickness, finer object imaging can be realized.

## Discussion

At this study we introduced a novel concept called the three-medium method to elucidate the FP resonance by utilizing the relevant theories in acoustic regime. We carried out analytical calculations over a wide frequency range (100–20,000 Hz), which confirmed the existence of a uniform unity transmission coefficient at FP resonance frequencies for both the evanescent and propagating waves. As the evanescent wave has a higher parallel wave momentum, it contains finer image information. Hence, by retrieving the evanescent wave along with the propagating wave on the image plane, it was possible to break through the diffraction limit. Subsequently, numerical and experimental analyses were performed which confirmed the existence of deep-subwavelength imaging. As an evanescent wave decays exponentially with travelling distance, after leaving the lens, it instantly began to fade. Therefore, an image can only be realized in close proximity to the lens.

However, two point sources were considered, and the distance between them was on the deep-subwavelength scale. Without a lens, the sources were indistinguishable, as verified both numerically and experimentally. However, when a metalens was employed, as expected, both sources were clearly distinguishable on the image plane. The maximum numerical resolution was found to be λ/56.53 for *h* = 212 mm. The experimental results corroborated the findings of the numerical analyses and provided evidence for the highest resolution of λ/56.53.

During lens fabrication, consideration should give to the λ/*a* ratio. As the transmission equation will be true until the ratio as very high, it is highly unlikely that all the higher modes of the lens can participate in imaging. At higher frequencies, the ratio will become smaller, and in turn, it will no longer conform to the assumption c_2_ = c_0_ for obtaining the transmission Eq. (2). However, during lens fabrication for pragmatic applications, it might be difficult to maintain a very high ratio. In addition, as the periodicity (*Λ*) acts like a pixel of the image, by reducing *Λ*, the image quality can be enhanced. Therefore, in future, we intend to deduce the lowest possible λ/*a* ratio empirically and optimize *Λ* to provide a framework to design FP lenses for high-frequency application.

The two-speaker experiments were performed carefully because if the distance between the speaker and lens is not same for each speaker, detecting the sources on the image plane may be difficult. This could occur because the evanescent wave, as discussed earlier, decays exponentially with distance. Therefore, single source study can be used to obtain insight into the highest possible resolution. However, note that with more speakers, it might not be possible to obtain the same resolution owing to interference and noise. Here, we considered the FWHM range provided by the lens to be the half power region in which the SPL drops to 3 dB from the peak. It was found that increasing the lens thickness enhanced the achieved resolution. Due to unavoidable noise, in the case of the experimental results, the obtained FWHM range was not as small as that predicted by the numerical analysis. However, both the experimental and numerical results provided consistent trends with regard to resolution versus thickness.

In short, we provided a fresh perspective to explain the FP-based lens. Further, we supported our theoretical exploration using FEM simulations and experiments. With careful design, we achieved the highest resolution ever reported for subwavelength imaging. Finally, we expect the study represented here will open new avenue in the area of FP lens and, for the deep-subwavelength imaging capability of such lens to facilitate applications in medical imaging, biometrics, and non-destructive testing.

## Methods

### Lens fabrication

Three lenses were fabricated with an array of square-shaped parallelepiped apertures using a 3D printer. The lenses with thicknesses *h* = 40 and 80 mm were fabricated using an Object 30 Pro 3D printer (Stratasys Company) and transparent VeroClear material, which consisted of transparent thermoplastic polymethyl methacrylate (PMMA). Owing to its larger thickness, the lens with *h* = 212 mm was fabricated using a stereolithographyapparatus (SLA) with a Kings 3D laser printer and a photocurable resin material. All three lenses consisted of a 2D array of 30 $\times { }$ 10 holes in the y- and z-directions, respectively. The length of the side of the square hole (*a*) and the periodicity (*Λ*) were 1.0 and 1.5 mm, respectively.

### Simulation

The numerical data were obtained via the FEM-based commercial software COMSOL Multiphysics. Simulations were performed by applying Acoustic Pressure, Frequency Domain (.acpr) physics. To reduce the model size, the perfectly matched layer (PML) was employed surrounding the model with a 20 mm thickness. Parallelepiped air holes with side length *a* = 1.0 mm and thicknesses *h* = 40, 80, or 212 mm were placed individually between two air blocks with periodicity *Λ* = 1.5 mm in a 2D array of 20 × 7 blocks in the y- and z- directions, respectively (Fig. 4). The array of hole blocks acts as a lens to perform subwavelength imaging. Monopole point sources used to generate the sound pressure were placed in front of the lens. To reduce the computational time and complexity, only the air part was meshed. A 1D array of data points along the y-direction behind the lens was created to scan the SPL. The separation between two adjacent points was 0.5 mm.

### Experimental setup

As shown in Fig. 6a, two micro-speakers (DTEC-30008-000, diameter of speaker opening = 1.5 mm) placed in front of the metalens were excited by a function generator (Tektronix, AFG 31000) with a 1 V_p-p_ sinusoidal wave. Behind the lens, a microphone (G.R.A.S. 26 CB, diameter = ¼ʺ) was placed that could be moved along the y-axis by a stepper motor to scan the SPL. The maximum resolution of the motor was 1 µm and the sensitivity of the microphone was 4 mV/Pa. As shown in Fig. 6a,b, the analogue data received from the microphone were converted to digital data by a four-channel National Instrument (NI) data acquisition (DAQ) card (Model NI 9234). Finally, to derive the SPLs, the digital data were manipulated using the LabVIEW-based software NI Signal Express 2013. Note that the reference pressure was fixed to 20 µPa to represent the data in decibels.

## Data Availability

The datasets generated and/or analyzed during the current study are available from the corresponding author on reasonable request.
